# The physical significance of acoustic parameters and its clinical significance of dysarthria in Parkinson’s disease

**DOI:** 10.1038/s41598-020-68754-0

**Published:** 2020-07-16

**Authors:** Shu Yang, Fengbo Wang, Liqiong Yang, Fan Xu, Man Luo, Xiaqing Chen, Xixi Feng, Xianwei Zou

**Affiliations:** 1 College of Medical Information Engineering, Chengdu University of Traditional Chinese Medicine, Chengdu, 610072 Sichuan China; 2 Department of Public Health, Chengdu Medical College, Chengdu, 610500 Sichuan China; 3 Department of Rehabilitation, First Affiliated Hospital of Chengdu Medical College, Chengdu, 610500 Sichuan China; 4 Department of Pharmacy, Chengdu Medical College, Chengdu, 610500 Sichuan China; 5 Department of Neurology, First Affiliated Hospital of Chengdu Medical College, Chengdu, 610500 Sichuan China

**Keywords:** Biophysics, Neurology

## Abstract

Dysarthria is universal in Parkinson’s disease (PD) during disease progression; however, the quality of vocalization changes is often ignored. Furthermore, the role of changes in the acoustic parameters of phonation in PD patients remains unclear. We recruited 35 PD patients and 26 healthy controls to perform single, double, and multiple syllable tests. A logistic regression was performed to differentiate between protective and risk factors among the acoustic parameters. The results indicated that the mean *f0*, max *f0*, min *f0*, jitter, duration of speech and median intensity of speaking for the PD patients were significantly different from those of the healthy controls. These results reveal some promising indicators of dysarthric symptoms consisting of acoustic parameters, and they strengthen our understanding about the significance of changes in phonation by PD patients, which may accelerate the discovery of novel PD biomarkers.

## Introduction

Parkinson’s disease (PD), a chronic, progressive neurodegenerative disorder with an unknown etiology, is associated with a significant burden with regards to cost and use of societal resources^1,2^. More than 90% of patients with PD suffer from hypokinetic dysarthria^3^. Early in 1969, Darley et al. defined dysarthria as a collective term for related speech disorders. The classification of dysarthria includes flaccid dysarthria, spastic dysarthria, ataxic dysarthria, hypokinetic dysarthria, hyperkinetic dysarthria, unilateral upper motor neuron dysarthria and mixed dysarthria^4^. The speech abnormalities of patients with PD are collectively termed hypokinetic dysarthria (HKD). These speech flaws are typically characterized by increased acoustic noise, a reduced intensity of voice, harsh and breathy voice quality, increased voice nasality, monopitch, monoloudness, speech rate disturbances, the imprecise articulation of consonants, the involuntary introduction of pauses, the rapid repetitions of words and syllables and sudden deceleration or acceleration in speech.

Speech impairments are caused by impaired speech mechanisms during any of the basic motor processes involved in speech performance^5^. The neuromotor speech sequence activates the muscles of the pharynx, tongue, larynx, chest and diaphragm through subthalamic secondary pathways. The anatomical substrate that could result in the abnormalities of PD phonetics may be reduced by the poor coordination of the sound-making muscles^6^. Usually, the stiffness of the laryngeal muscle tissue, which results in an increased hardness of the vocal cords, affects the closure of the vocal cords and increases the muscle tone^7^. Moreover, due to the decreased controllability of the diaphragm, the pneumatic input of the lungs to the larynx and the lung capacity decrease significantly^8^. Fortunately, dysphonia in PD has recently received abundant attention^9^.

There are three dominant factors that affect dysphonia: aerodynamic deficits, inefficient vibratory function, and weak muscle activity. First, the high phonatory resistance in PD patients may be due to a significant increase in their estimated subglottic pressure and a reduction in their phonatory airflow^10^. Previously tested patients had insufficient exhalation volume per syllable, and reductions in lung volume are associated with increased sound severity^11^. Second, the abnormal vibration of the vocal cords also affects dysarthria. Laryngopharyngeal involvement manifests as vocal fatigue, vocal breaks, tremor and the inability to make sound^12,13^. These manifestations may lead to the inadequate or excessive closing of the vocal cords and irregular or asymmetrical vocal fold motion during phonation^14,15^. Finally, dysarthria is the result of involuntary movements that are variable and irregular in nature^5^.

The primary manifestations of PD are tremor^16^, rigidity^17^, bradykinesia^18^, postural instability^19^, slowness of movement, hyposmia^20^, sleep disorders^21^ and changes in sound during speech^22^. A noticeable tonal change can occur during PD progression; however, it is often ignored. PD patients may present characteristics such as sound quality change^23^, poor articulation^24^, trembling or hoarseness^25,26^, increased frequency and jitters^7,27^, lower tone^28^, decreased rhythm^29^, lack of emotional expression^25^, and tonal changes^29^. However, the quantification of the tonal change remains ambiguous.

For this reason, the early diagnosis of PD with accurate, reliable and unbiased predictive models is crucial for PD patients^30,31^. Previous studies revealed that vocal changes, including poor articulation, trembling or hoarseness, frequency changes, degraded sound quality, lower tone, decreased rhythm, a lack of emotional expression and tonal changes, are important manifestations showing the early development of PD. Muscular hypertension reduces the controllability of vocal cord vibrations and allows insufficient airflow into the lungs for vocal sounds to proceed smoothly^32^. However, our understanding of the link between physical features and clinical significance remains unclear. Consequently, we aimed to clarify the current state of the voice features during the early stage of developing dysarthria in PD. Moreover, protective factors and risk factors were distinguished from these acoustic parameters.

## Methods

### Participants

Two groups of participants (PD patients and controls) were recruited for this study from January to August 2019. For the idiopathic PD group, 35 participants (21 male and 14 female patients) were recruited. According to the UK Parkinson’s Disease Society Bank Criteria, they had been diagnosed with idiopathic PD in our neurology department prior to the recruitment interval. Furthermore, the dysarthria level in the PD patients was assessed using the Voice Handicap Index (VHI-30)^33^. All the participants were assessed by investigator inquiry for their living habits in terms of alcohol consumption and smoking. Each PD patient was assessed using the Hoehn–Yahr scale (H&Y) and the Unified Parkinson’s Disease Rating Scale Motor Score (UPDRS III). A trained neurologist conducted the entire assessment process. Twenty-six age- and sex-matched healthy participants were recruited for the control group. During the first three months of the study, the participants participated in no other clinical trials. The exclusion criteria for all the participants were as follows: (1) a history of a communication or neurological disorder; (2) throat disease, such as pharyngitis, laryngitis, or laryngeal tumors; (3) severe mental disorder or cognitive impairment, which may hinder speech; (4) psychotic or systemic major illness; (5) clinical problems such as aphasia and severe dysarthria; (6) history of acute stroke, sports injury, or mental disorder; (7) long-term use of systemic treatment methods that affect sound detection; (8) inability to complete the study tasks accurately; and (9) participation in other rehabilitation projects. All the patients stopped taking levodopa on the morning of the sound test, they continued to take other anti-Parkinson's drugs and were still in the “ON” phase. Therefore, the sound test was not performed during the “Off” phase, when the patients had severe motor symptoms.

### Voice recordings and data preparation

The clinician guided and oversaw the quality control of the voice test. Prior to this test, every participant was thoroughly introduced to the general voice test workflow by the clinician. Therefore, all the participants could cooperate to complete the entire task smoothly. There was no time limit for any item until the inspector was satisfied with the subject’s performance.

Vowels, including /a/, /o/, and /e/, are often used in the phonetic test, which involved the movement of the utterance organ in various positions^34^. Here, every syllable consisted of a vowel, and the quantity of syllables depended on the quantity of Chinese characters, namely, a single syllable had only one Chinese character, a double syllable had a double Chinese character, and multiple syllables had no more than 5 Chinese characters. There were 12 single syllable samples, 8 double syllable samples and 6 multiple syllable samples for every participant. Sound recordings were performed in a low-noise (< 50 dB) room. An acoustic recording pen (Sony, Japan) was held 30 cm from the subject's mouth to record their sound. It was important to ensure that the subject maintained their normal tone and loudness in a relaxed state while recording. All the participants were subject to the guidelines of the clinician. If the subject felt tired, the test was paused until he/she felt comfortable completing the remainder.

### Acoustic parameter extraction

A total of 1,400 sound clips were collected. The duration of one clip (single syllable, double syllable, multiple syllables) is approximately 4–7 s. The duration of the entire recording process was an average of 12 min per candidate for one test. Subsequently, 12 acoustic parameters were extracted using a customized MATLAB script. The acoustic parameters included (a) start *f0* (Hz), (b) mean *f0* (Hz), (c) mid *f0* (Hz), (d) minimum *f0* (Hz), (e) maximum *f0* (Hz), (f) end *f0* (Hz), (g) slope from the maximum *f0* to the end of the call (slope M-E; Hz/s), (h) slope from the start *f0* of the call to the maximum *f0* (slope S-M; Hz/s), (i) median intensity (Hz), (j) duration of speaking (seconds) (k) harmonics-to-noise ratio (HNR, Hz) and (l) jitter (the absolute *f0* difference between consecutive *f0* measurements/the average period).

### Statistical analysis

All the data were stored in Excel files. The data are represented as the means ± s. dev. All the analyses were performed with STATA 15.0 (StataCorp. 2017. Stata Statistical Software: Release 15. College Station, TX: StataCorp LLC). In brief, the chi-square test was used to analyze the differences in the demographic distribution by sex, profession, alcohol consumption, smoking habit and educational level. A Student’s t-test was conducted to assess whether there was an age difference between the two groups. If the data were not normal, the acoustic parameters between the two groups were compared using a Mann–Whitney test. If the data were normally distributed, the Student's t-test was employed. A logistic regression analysis was performed to differentiate the protective factors and risk factors from among the acoustic parameters. To test the collinearity of the regression model, the Pearson correlation coefficients were calculated among the seven parameters in the model. The absolute value of the coefficient between these parameters was very small, with the largest being 0.242, and most of them were less than 0.1. Additionally, the meaning of the parameters is completely different, and thus it is not appropriate to use the dimensionality reduction method to solve the collinearity problem. In addition, the prediction effect is reasonable and rich with professional significance. Therefore, this weak collinearity can be ignored. Spearman's rank correlation coefficient was used to determine the correlation of the acoustic parameters with the H&Y, UPDRS III and VHI-30. A *P *value of < 0.05 was considered significant.

## Results

There was no significant difference in the age distribution between the two groups (*t* = 0.5305, *df* = 59, *P* = 0.7011). No statistically significant sex difference was found between the two groups (*χ*^2^ = 1.874, *df* = 1,* P* = 0.171). In addition, no significant differences were found in the patient alcohol consumption or smoking habits. However, a difference in the distribution of professions was found between the two groups (Fisher’s exact test, *χ*^2^ = 6.2674,* df* = 2,* P* = 0.044). Similarly, there was a significant difference in educational levels between the two groups (*χ*^2^ = 8.8961,* df* = 3,* P* = 0.03). The average H&Y and UPDRS III scores of the PD group were 2.60 ± 0.81 and 35.60 ± 20.39, respectively (Table 1).

**Table 1:** Baseline characteristics of the participants.

Variables	PD	Control	Statistics
N	35	26	
Age*	67.57(8.78)	66.46(7.02)	*P* = 0.70
Sex^#^ (M/F)	21/14	11/15	*P* = 0.17
Duration of disease	4.59 (3.75)	–	
VHI-30	22.46(10.30)	–	
H&Y	2.60(0.81)	–	
UPDRS III	35.60(20.39)	–	
**Profession**^#^			*P* = 0.04
Retired	10	1	
Farmer	17	18	
Worker	8	7	
Alcohol consumption^#^ (N/Y)	27/8	23/3	*P* = 0.26
Smoker^#^ (N/Y)	26/9	23/3	*P* = 0.17
**Education**^#^			*P* = 0.03
Primary school	19	23	
Middle school	10	1	
High school	5	2	
Master’s	1	0	

*H*&*Y* Hoehn and Yahr stage, *UPDRS III* Unified Parkinson’s Disease Rating Scale III

^#^Chi-square test, *Student's t-test

With the aim of reducing the effect from the sex, Table 2 compares the average acoustic parameters between the PD group and the control group for different sexes. The mean *f0* among the three syllable tests was lower in the PD group than in the control group among males. Among these findings, the two groups of single-syllable tests displayed significant differences (*P* < 0.05). The min *f0* was significantly different in both the double syllable test and the multiple syllable test in males between double groups (*P* < 0.05). Regarding the female patients, the single and double syllable tests presented significant differences between the double groups (*P* < 0.05). Moreover, the max *f0* was significantly lower in the PD group than in the control group among the males. For female patients, a significant difference was found only in the single syllable test between double groups. Interestingly, female PD patients presented significantly higher results than the control. By contrast, this finding differs from that of the male patients, and we believe that the reasons are the sex hormone difference and anatomical structural differences between the sexes. Similarly, the end *f0* in the single syllable and double syllable tests, the slope S-M in the single syllable test and the median intensity in the multiple syllable test of the PD female patients were significantly higher than those of the controls. In the male double syllable and multiple syllable test, the duration of the PD group was significantly shorter than that of the control group. The jitter results showed statistical differences between groups in the male double syllable test only.

**Table 2:** Comparison of mean values in sound parameters between PD and control patients.

Acoustic parameter	Sex	Male						Female					
	Group	Control		PD				Control		PD			
	Syllable	n	Mean ± SD	n	Mean ± SD	t	*P*	n	Mean ± SD	n	Mean ± SD	t	*P*
Start *f0* (Hz)	Single	133	178.64 ± 89.33	180	169.48 ± 70.82	1.012	0.312	180	168.92 ± 83.82	154	153.63 ± 74.62	1.747	0.082
	Double	87	186.59 ± 91.92	120	174.54 ± 76.67	1.026	0.306	120	173.88 ± 84.72	104	167.17 ± 78.93	0.610	0.543
	Multiple	64	179.40 ± 94.64	90	179.62 ± 84.26	– 0.015	0.988	90	175.35 ± 86.75	78	165.94 ± 83.08	0.715	0.475
Mean *f0* (Hz)	Single	133	170.29 ± 23.13*	180	164.81 ± 23.83*	2.035	0.043	180	165.8 ± 24.57	154	167.62 ± 20.55	– 0.728	0.467
	Double	87	169.27 ± 22.44	120	166.22 ± 23.87	0.929	0.354	120	168.13 ± 20.36	104	168.68 ± 21.3	– 0.200	0.842
	Multiple	64	172.65 ± 22.98	90	171.04 ± 23.5	0.423	0.673	90	167.92 ± 18.68	78	172.84 ± 21.34	– 1.596	0.112
Mid *f0* (Hz)	Single	133	163.85 ± 81.68	180	166.96 ± 78.15	– 0.341	0.733	180	172.75 ± 81.78	154	168.26 ± 85.88	0.489	0.625
	Double	87	167.43 ± 71.58	120	170.83 ± 82.39	– 0.31	0.757	120	172.58 ± 68.59	104	185.4 ± 71.9	– 1.364	0.174
	Multiple	64	175.67 ± 65.49	90	180.34 ± 78.23	– 0.391	0.697	90	184.87 ± 82.95	78	181.67 ± 67.19	0.271	0.786
Min *f0* (Hz)	Single	133	50.24 ± 0.50	180	50.24 ± 0.51	0.082	0.935	180	50.20 ± 0.52*	154	50.09 ± 0.23*	2.437	0.015
	Double	87	50.15 ± 0.35*	120	50.31 ± 0.53*	– 2.402	0.017	120	50.25 ± 0.64*	104	50.09 ± 0.20*	2.464	0.014
	Multiple	64	50.38 ± 0.73*	90	50.18 ± 0.33*	2.210	0.029	90	50.24 ± 0.60	78	50.12 ± 0.30	1.589	0.114
Max *f0* (Hz)	Single	133	395.18 ± 12.38*	180	389.27 ± 17.51*	3.327	0.001	180	388.81 ± 21.00*	154	393.80 ± 11.04*	– 2.653	0.008
	Double	87	393.91 ± 12.50*	120	386.73 ± 17.90*	3.216	0.002	120	390.89 ± 16.34	104	391.34 ± 14.22	– 0.222	0.825
	Multiple	64	397.76 ± 7.32*	90	390.92 ± 15.82*	3.218	0.002	90	395.40 ± 10.99	78	393.67 ± 12.36	0.959	0.339
End *f0* (Hz)	Single	133	246.33 ± 102.11	180	244.25 ± 98.7	0.182	0.856	180	239.21 ± 95.14*	154	273.15 ± 90.38*	– 3.325	0.001
	Double	87	243.06 ± 100.86	120	247.46 ± 100.88	– 0.310	0.757	120	241.52 ± 100.82*	104	268.31 ± 97.19*	– 2.017	0.045
	Multiple	64	232.86 ± 93.12	90	248.65 ± 106.8	– 0.953	0.342	90	252.59 ± 87.49	78	277.14 ± 92.6	– 1.766	0.079
Slope M-E (Hz/s)	Single	131	– 104,311.52 ± 396,649.63	173	– 92,991.59 ± 258,141.83	– 0.301	0.764	179	– 65,088.06 ± 174,637.86	151	– 103,680.7 ± 402,912.44	1.159	0.247
	Double	86	– 189,942.18 ± 565,674.53	118	– 311,437.95 ± 920,745.92	1.083	0.280	119	– 182,311.76 ± 748,104.78	103	– 95,433.15 ± 274,063.43	– 1.115	0.266
	Multiple	64	– 94,861.61 ± 388,677.88	90	– 162,047.38 ± 432,150.88	0.991	0.323	89	– 79,175.53 ± 324,979.16	77	– 55,075.06 ± 171,983.45	– 0.584	0.560
Slope S-M (Hz/s)	Single	130	– 5,132.11 ± 469,415.76	178	– 10,348.38 ± 242,847.61	0.127	0.899	173	– 42,172.1 ± 325,913.7*	154	32,581.48 ± 338,176.18*	– 2.034	0.043
	Double	85	5,179.32 ± 54,268.93	119	45,252.06 ± 377,235.86	– 0.972	0.332	119	– 33,834.21 ± 980,782.41	104	10,345.3 ± 72,610.16	– 0.458	0.647
	Multiple	63	25,357.42 ± 197,497.6	89	424.63 ± 27,987.22	1.176	0.241	86	95,398.45 ± 838,470.75	77	4,385.96 ± 50,836.81	0.951	0.343
Median intensity (Hz)	Single	133	157.99 ± 30.27	180	158.4 ± 37.09	– 0.105	0.917	180	155.72 ± 37.01	154	159.53 ± 41.65	– 0.885	0.377
	Double	87	154.37 ± 25.56	120	161.57 ± 34.27	– 1.654	0.100	120	160.2 ± 31.1	104	167.9 ± 38.21	– 1.663	0.098
	Multiple	64	156.52 ± 31.76	90	166.57 ± 34.01	– 1.859	0.065	90	157.52 ± 30.64*	78	170.76 ± 37.14*	– 2.531	0.012
Duration (s)	Single	133	5.80 ± 0.77	180	5.67 ± 0.59	1.775	0.077	180	5.79 ± 0.96	154	5.96 ± 0.69	– 1.844	0.066
	Double	87	6.24 ± 0.61*	120	5.90 ± 0.63*	3.897	0.000	120	6.15 ± 1.06	104	6.39 ± 0.82	– 1.916	0.057
	Multiple	64	6.34 ± 1.15*	90	5.98 ± 0.93*	2.095	0.038	90	6.33 ± 1.34	78	6.29 ± 1.17	0.162	0.871
HNR (Hz)	Single	133	– 5.93 ± 2.65	180	– 5.63 ± 2.64	– 0.976	0.330	180	– 5.81 ± 2.55	154	− 5.57 ± 2.92	− 0.794	0.428
	Double	87	− 5.92 ± 3.25	120	− 5.09 ± 3.41	− 1.779	0.077	120	− 5.38 ± 2.83	104	− 5.66 ± 2.39	0.792	0.429
	Multiple	64	− 5.51 ± 2.93	90	− 5.39 ± 3.05	− 0.247	0.805	90	− 5.61 ± 2.70	78	− 5.63 ± 2.85	0.048	0.962
Jitter	Single	133	2.62E−03 ± 5.44E−04	180	2.51E−03 ± 4.46E−04	1.886	0.060	180	2.65E−03 ± 5.71E−04	154	2.68E−03 ± 3.82E−04	−0.579	0.563
	Double	87	2.55E−03 ± 5.61E−04*	120	2.36E−03 ± 4.37E−04*	2.637	0.009	120	2.48E−03 ± 5.02E−04	104	2.48E−03 ± 3.23E−04	0.031	0.975
	Multiple	64	2.54E−03 ± 5.93E−04	90	2.44E−03 ± 5.25E−04	1.045	0.298	90	2.58E−03 ± 5.03E−04	78	2.53E−03 ± 4.56E−04	0.722	0.472

Student's t-test, *P < 0.05

*n* The number of sound clips

Subsequently, a logistic regression analysis was performed to determine the protective and risk factors among the acoustic parameters (Table 3). Specifically, Z values of less than 0 signify protective factors, including sex, alcohol consumption, start *f0*, min *f0*, max *f0*, slope S-M, jitter and duration. The other acoustic parameters were risk factors, including the age, smoking habit, education, end *f0*, HNR and median intensity.

**Table 3:** Regression analysis result of acoustic parameters among all participants.

Variables	Odds ratio	Std. Err	z	*P*	95% CI
Age	1.013748	0.0076428	1.81	0.07	[0.9988786, 1.028839]
Sex	0.6787941	0.0845371	− 3.11	0.002	[0.5317768, 0.8664566]
Alcohol consumption	0.6615954	0.1423631	− 1.92	0.055	[0.43394, 1.008684]
Smoking	2.852004	0.5308268	5.63	0	[1.980255, 4.107517]
Education	1.030776	0.0281676	1.11	0.267	[0.9770207, 1.087489]
Start *f0 *(Hz)	0.8132798	0.0968599	− 1.74	0.083	[0.6439671, 1.027108]
Min *f0 *(Hz)	0.9224733	0.1121296	− 0.66	0.507	[0.7269214, 1.170631]
Max *f0 *(Hz)	0.5067264	0.0590985	− 5.83	0	[0.4031805, 0.6368652]
End *f0 *(Hz)	1.743581	0.202403	4.79	0	[1.388771, 2.189039]
Slope S-M (Hz/s)	0.9341043	0.106015	− 0.6	0.548	[0.7478067, 1.166813]
Median intensity (Hz)	1.685214	0.2169086	4.05	0	[1.309467, 2.16878]
Duration (s)	0.7583914	0.0876285	− 2.39	0.017	[0.6047016, 0.9511427]
HNR (Hz)	1.156047	0.1336188	1.25	0.21	[0.9217043, 1.449972]
Jitter	0.9708554	0.1188046	− 0.24	0.809	[0.7638219, 1.234005]

In Table 4, the Spearman's rank correlation coefficient was used to determine the correlation of the acoustic parameters with the H&Y, UPDRS III and VHI-30. The single most striking observation to emerge from the data comparison was that the mean f0 was negatively correlated with the VHI-30 in the single and double syllable tests (*P* < 0.05, R < 0). Namely, the mid f0 was inversely related to the VHI-30 in the double syllable tests. Additionally, the max f0 was negatively correlated with the VHI-30 in monosyllable and multisyllable PD patients. The correlation between the end f0 and the H&Y was statistically significant in the double and multiple syllable tests (*P* < 0.05). In addition, these factors showed a negative correlation (R < 0). Moreover, a positive correlation was found between the slope M-E and UPDRS III in the single syllable test (*P* < 0.05, R > 0). The slope M-E of the single and double syllable tests was negatively correlated with the VHI-30. Interestingly, the median intensity and duration were significantly positively correlated with the H&Y and UPDRS III in the single syllable results (*P* < 0.05, R > 0). However, all the syllables of median intensity were related to the VHI-30. Furthermore, the HNR was negatively correlated with the UPDRS III in the multiple syllable findings (*P* < 0.05, R < 0). Likewise, the jitter showed a significant negative correlation with the H&Y in the single syllable test (*P* < 0.05, R < 0). The correlation between the jitter and the VHI-30 was present in the single and multiple syllable results.

**Table 4:** Correlation between clinical severity of PD and acoustic parameters.

Acoustic parameters	Syllable	H&Y		UPDRS III		VHI-30	
		R	*P*	R	*P*	R	P
Start *f0* (Hz)	Single	0.0363	0.5083	0.0734	0.1809	− 0.0134	0.8070
	Double	− 0.0080	0.9049	0.0934	0.1637	0.0186	0.7817
	Multiple	0.0533	0.4930	− 0.0274	0.7246	− 0.0307	0.6929
Mean *f0* (Hz)	Single	0.0878	0.1092	0.0557	0.3104	− 0.2435*	0.0000*
	Double	− 0.0382	0.5693	0.0738	0.2712	− 0.2198*	0.0009*
	Multiple	− 0.0513	0.5089	0.0425	0.5847	− 0.0778	0.3163
Mid *f0* (Hz)	Single	0.0264	0.6311	0.0760	0.1661	− 0.0422	0.4425
	Double	0.0396	0.5557	0.0464	0.4897	− 0.1512*	0.0236*
	Multiple	− 0.0678	0.3822	− 0.0564	0.4675	− 0.1295	0.0944
Min *f0* (Hz)	Single	0.0022	0.9686	0.0600	0.2742	− 0.0725	0.1861
	Double	0.0119	0.8593	0.0752	0.2623	− 0.0626	0.3510
	Multiple	− 0.0043	0.9561	0.1034	0.1823	− 0.1038	0.1805
Max *f0* (Hz)	Single	0.0357	0.5152	− 0.0102	0.8521	− 0.1485*	0.0066*
	Double	− 0.0540	0.4211	− 0.0563	0.4015	− 0.1444*	0.0308*
	Multiple	0.0776	0.3174	0.1400	0.0702	− 0.0985	0.2038
End *f0* (Hz)	Single	− 0.0368	0.5028	− 0.0914	0.0955	− 0.0835	0.1276
	Double	− 0.1361*	0.0419*	− 0.1021	0.1276	− 0.0976	0.1456
	Multiple	− 0.1744*	0.0238*	− 0.1175	0.1294	0.0262	0.7364
Slope M-E (Hz/s)	Single	− 0.1083	0.0514	0.0051*	0.0032*	− 0.1576*	0.0045*
	Double	− 0.1078	0.1099	− 0.0783	0.2462	− 0.1429*	0.0337*
	Multiple	− 0.1245	0.1089	− 0.0602	0.4398	− 0.1244	0.1093
Slope S-M (Hz/s)	Single	0.0757	0.1686	0.0051	0.9264	− 0.0988	0.0723
	Double	− 0.0110	0.8705	− 0.0335	0.6188	0.0679	0.3130
	Multiple	0.0656	0.4008	0.0951	0.2227	− 0.0195	0.8029
Median intensity (Hz)	Single	0.1864*	0.0006*	0.1467*	0.0072*	− 0.2046*	0.0002*
	Double	0.0073	0.9138	0.1024	0.1266	− 0.2114*	0.0015*
	Multiple	0.0138	0.8591	0.0659	0.3957	− 0.1925*	0.0124*
Duration (seconds)	Single	0.1839*	0.0007*	0.2111*	0.0001*	0.0035	0.9498
	Double	0.0744	0.2674	0.0846	0.2070	0.0593	0.3774
	Multiple	0.1342	0.0829	0.0834	0.2824	0.0544	0.4840
HNR (Hz)	Single	− 0.0057	0.9179	− 0.0607	0.2690	0.0113	0.8373
	Double	0.0289	0.6665	0.0564	0.4007	0.0360	0.5922
	Multiple	− 0.0966	0.2130	− 0.1820*	0.0182*	− 0.0465	0.5495
Jitter	Single	− 0.1686*	0.0020*	− 0.0373	0.4967	− 0.1150*	0.0357*
	Double	− 0.0609	0.3646	− 0.1048	0.1177	− 0.0995	0.1377
	Multiple	0.0402	0.6045	− 0.0316	0.6846	− 0.2080*	0.0068*

Spearman analysis, *P < 0.05.

## Discussion

Among the participants, the results showed a significant difference in the distribution of professions and education across both groups; a large proportion of participants were farmers, and a low educational level was noted for a larger proportion of patients than other education levels (see Table 1).

Our data revealed significant differences in the mean *f0*, min *f0*, max *f0*, duration and jitter in male participants between the two groups and significant changes in the min *f0*, max *f0*, end *f0*, slope S-M and median intensity in female participants between the two groups (see Table 2).

The vocal fold vibration frequency is known as the fundamental frequency. The PD group presented a lower start *f0*, lower max *f0*, higher mid *f0* and higher end *f0* than the control group. The start *f0*, min *f0*, max *f0* and end *f0* denote the start, minimum, maximum and end values of produced *f0* movement in the syllables, respectively. These variables can be used to describe the entire vocalization process. However, we found a significant difference from the healthy group only among the mean *f0* in male participants speaking a single syllable, min *f0* in both male and female participants and end *f0* in female participants speaking single and double syllables. Holmes et al. examined the correlates of PD voice disorders in the fundamental frequency (*f0*) of the pronounced vowels, and they found that the speaking *f0* of the PD patients was reduced^35^. The vocal cord vibration frequency, vocal cord status information related to voice quality and energy, muscle contraction, joint movement and sound intensity are provided to the CNS by somatosensors located in and near the larynx^36^. The stimulation of the superior laryngeal nerve, laryngeal mucosa or cartilage results in the reflexive contraction of laryngeal muscles. These reflections may be important for controlling the voice *f0*^37^.

Moreover, our data showed that *slope S-M* was significantly larger than the control group in female PD polysyllables. Slope S-M is the slope from the start to the maximum of the pronunciation, and slope M-E is the slope from the maximum to the ending. This factor measures the rate of airflow caused by the coordinated movement of lung muscles. In addition, our data revealed that the *median intensity* of male PD patients was higher than that of the control group in the overall syllable test. Similarly, the female PD patients also presented a higher median intensity than the controls. However, only during the multiple syllable test did female PD patients present significantly higher intensity than the control group. The median intensity represents the intensity of vocalization. Having a low voice is also one of the symptoms of PD patients. This characteristic is due to the stiffness of the larynx muscles in PD patients, which makes pronunciation difficult. In 2018, Abur et al. studied the loudness of pure tone between PD patients and a control group. The results showed that the average loudness growth slopes of the control and PD groups were not significantly different, while the tone perception and loudness of the PD patients decreased^38^. Unfortunately, these results are contrary to our findings. As PD patients worsen, they usually develop a low voice, especially during the middle and late stages of the disease^39^. In our study, most patients with PD had a low level of dysarthria and were in the early and middle stages of the disease. Therefore, the difference in sound intensity may be due to the different severity levels of the disease among PD patients.

Notably, we found that PD patients read the same sentence for a shorter *duration* than the controls. We found a significant difference in the duration in male participants speaking double and multiple syllables. The duration refers to the time the subject takes to read the same sentence. This measurement is related to speech rhythm and time organization^29^. The dopamine in the basal ganglia of PD patients is gradually depleted, which is the primary cause of muscle stiffness, and it changes the controllability of the larynx muscles^40^. Subsequently, muscle stiffness in the throat and pharynx can significantly affect the pronunciation speed and number of pauses^41^, indicating impaired speech rhythm and timing^29^. Furthermore, the muscle contraction intensity in the chest cavity and diaphragm is significantly reduced, which leads to a reduction in the airflow from the lungs through the vocal cords. Ultimately, the reduced airflow affects the vibration of the vocal cords, and the shape of the vocal cords affects the sound pressure threshold^8^. These interactions may reduce the time for vocalization. Similarly, Hammer et al. compared the air flow and acoustic parameters between PD patients and controls. Their results showed that PD patients presented a shorter syllable-speaking duration than the controls^11^. PD patients present several significant speech characteristics, including an increasing trend in the speech rate and a reduction in the total number of pauses. Our findings are consistent with these previously reported results.

In addition, the *HNR* is expressed as the degree of acoustic periodicity and is used to estimate the signal-to-noise ratio by calculating the autocorrelation of each cycle. The HNR can be used to correlate the laryngeal pathology and voice changes, indicating the hoarseness of the voice. The lower the value is, the higher the hoarseness of the sound. This value also reflects the muscle tone of its larynx^42,43^. Our results showed that the PD patients displayed lower HNR than the control subjects, but the difference was not statistically significant. Zwirner et al. found that the HNR was lower in PD patients than in controls, but no significant difference was found^44^. This finding is consistent with our research. The perturbation in frequency in successive vocal fold cycles is termed *jitter*, which may be related to tremors in the vocal cords of PD patients. Due to the lack of control over the vocal cord vibration cycle of the glottis, the jitter may change, which is usually found in neurological diseases^45^. Our results indicated that the jitter values were lower in PD patients than in control subjects, and there were significant differences between male participants speaking double syllables. As the jitter of the sound decreases, its periodicity also decreases, and a creakier voice will be produced^46^. The depletion of dopaminergic neurons in the substantia nigra pars compacta usually leads to muscle rigidity and changes in the muscle control of the larynx (phonatory subsystem), which may induce increased throat tension (which is physiologically related) and decreased verbal variability^47^. Nevertheless, Gamboa et al. found that both male and female PD patients had higher jitter values than control subjects. The increased jitter may be related to the perceived low tone, which should correspond to the real *f0*^48^.

The Z-values from the regression analysis indicated that the acoustic parameters of start *f0*, min *f0*, max *f0*, slope S-M, duration and jitter were less than zero, signifying that these parameters are positive factors (Table 3). Pathological voice tremor occurs when involuntary and rhythmical oscillatory movements are initiated in the vocal tract. These movements can induce rhythmic fluctuations in the fundamental frequency and amplitude of the voice^49^. These fluctuations are perceived as rhythmic fluctuations in pitch and loudness. For example, Midi et al. revealed that patients with PD had higher jitter, fundamental frequency and fundamental frequency variability than control subjects. These results indicated that the higher *f0* and *f0* variations in PD patients are generally attributable to the increased stiffness of the vocal folds because of the rigidity of the laryngeal musculature^7^. Moreover, Alexander et al. analyzed the acoustic characteristics of PD speech before and after PD patients took a medication, and they revealed a higher fundamental frequency (*f0*) variability in vowels and mean *f0* but a lower intensity range in PD patients than in the controls^50^.

Conversely, the Z-values from the regression analysis indicated that the acoustic parameters of end *f0*, HNR and the median intensity were greater than zero, signifying that they are negative factors. End *f0* represents the frequency of vocal fold vibration at the end of speech, which indicates the voice change trend from the maximum frequency to the ending frequency. There was a significant difference in the end *f0* between the two groups of female participants. Interestingly, the results of the logistic regression analysis revealed that the end *f0* was a negative factor. Moreover, the absolute HNR was smaller in the PD group than in the control group except in the double- and multiple-syllable tests in the female PD group. Yumoto et al. demonstrated that lower absolute HNR values correspond to a greater proportion of noise. This finding suggests that a lower HNR represents a larger proportion of noise. Yumoto et al. also showed that HNR is an indicator of the degree of hoarseness^43^. Rusz et al. showed that patients with PD had lower HNR than the control subjects, which may be clinically interpreted as hypophonia, voice hoarseness, or tremolo^51^. It is worth noting that the median intensity was a protective factor in the logistic analysis, which indicates that the lower vocalization in PD corresponds to worse PD severity. Namely, the healthy controls presented louder speech. Rusz et al. assessed the extent of vocal impairment in PD patients and healthy controls, and the results revealed that PD patients have an overall lower speech intensity level, insufficient intensity range, and intensity variations during speech production^25^.

Our results showed that the acoustic parameters of end *f0*, HNR and jitter were negatively correlated with the clinical severity of PD. The slope M-E, median intensity and duration were positively correlated with the severity. Bayestehtashk et al. conducted three tasks, namely the sustained phonation task, the diadochokinetic task and a reading task with 168 PD patients, and they used a time-varying harmonic model of speech to capture clues related to pitch more accurately, including the jitter and shimmer. The results show that the severity of the disease can be inferred from speech, with an average absolute error of about 5.5, explaining 61% of the variance^52^. Similarly, Asgari et al. showed that it is possible to predict the severity of the disease by extracting voice information from PD patients (time domain, spectrum domain, cepstrum domain, HNR, and jitter)^53^. This finding also reflects the correlation between the voice information of these PD patients and the severity of the disease.

Our research described the current state of voice features at either an early stage of PD or an early stage of developing dysarthria. In addition, we discovered the significance of the physical and clinical aspects of the acoustic parameters. The quality of speech changes is universal in PD patients during disease progression.

## Conclusion

The mean *f0*, max *f0*, min *f0*, jitter, duration and median intensity of speaking in PD patients were significantly different from those of the healthy controls. The end *f0*, slope M-E, median intensity, duration, HNR and jitter are related to the clinical severity of PD. In addition to these parameters, the mean *f0*, mid *f0*, and max *f0* are negatively related to the VHI-30. These changes may strengthen public awareness of PD disease progression.

### Limitation

First, although the patients stopped taking levodopa on the morning of the sound test, they continued to take other anti-Parkinson's drugs and was still in the “ON” phase. Therefore, the sound test was not measured during the “Off” phase when the patient had severe motor symptoms. Second, we did not perform a further comparative analysis on the speech of the PD patients at different stages of disease development. Third, the variation in the experimental data may be affected by the current situation of the participant in terms of age, sex and medication regimen. Moreover, we have not yet evaluated voice tremors in PD patients using the related scales.

### Ethics approval and consent to participate

The Institute's Institutional Review Board and Ethics Committee at the First Affiliated Hospital of Chengdu Medical College approved this study. All experiments were performed in accordance with relevant guidelines and regulations. Written informed consent was provided by all participants.

### Consent for publication

Yes.

## Data Availability

All the data will be made available upon reasonable request.

## Abbreviations

PD: Parkinson’s disease
HKD: Hypokinetic dysarthria
VHI-30: Voice Handicap Index
H&Y: Hoehn–Yahr scale
UPDRS III: Unified Parkinson’s Disease Rating Scale Motor Score
